# Molecular profiles to biology and pathways: a systems biology approach

**DOI:** 10.1186/s40880-016-0112-4

**Published:** 2016-06-16

**Authors:** Steven Van Laere, Luc Dirix, Peter Vermeulen

**Affiliations:** Translational Cancer Research Unit, Center for Oncological Research, Faculty of Medicine and Health Sciences, University of Antwerp, Oosterveldlaan 24, Wilrijk, 2610 Antwerp, Belgium

**Keywords:** Systems biology, Data integration, Pathways, Networks, Topology

## Abstract

Interpreting molecular profiles in a biological context requires specialized analysis strategies. Initially, lists of relevant genes were screened to identify enriched concepts associated with pathways or specific molecular processes. However, the shortcoming of interpreting gene lists by using predefined sets of genes has resulted in the development of novel methods that heavily rely on network-based concepts. These algorithms have the advantage that they allow a more holistic view of the signaling properties of the condition under study as well as that they are suitable for integrating different data types like gene expression, gene mutation, and even histological parameters.

For many researchers worldwide, unravelling the biology of human tumors, either primary or metastatic, is a daily practice. The development of high-throughput technologies, such as microarrays and next-generation sequencing, has greatly accelerated our understanding of the complex molecular underpinnings of cancer development and progression. Big sequencing consortia like The Cancer Genome Atlas and the International Cancer Genome Consortium provide the research community with an unprecedented wealth of genomic, epigenomic, transcriptomic, and proteomic details of various tumor and cell types [1–8]. The recent landscape of published articles represents only the initial analyses, and answers to many other important questions may well be buried deep inside the reported data.

Many cancer profiling experiments have the common goal of identifying the signal transduction pathways and processes that characterize tumor biology, with the ultimate aim of discovering novel targets for treatment. The current mass production of molecular cancer profiles has spurred the development of novel tools particularly designed for this purpose. Most of these tools build on the concept of gene set enrichment analysis (GSEA), which evaluates whether the overlap between two gene sets is greater than that expected by chance [9]. Analyzed gene sets represent lists of significantly mutated or overexpressed genes on the one hand and lists of gene-associated pathways or processes on the other hand. The latter are based on prior knowledge gained through decades of basic and translational research and can be found in various publically available databases, such as the Gene Ontology, the Kyoto Encyclopedia of Genes and Genomes, and the Biocarta.

However, novel biological insights have increasingly called into question the classical representation of processes and particularly pathways as hierarchically structured as well as mostly linear diagrams of protein–protein interactions that are sharply and precisely delineated from the broader cellular transduction network. Instead, the now-prevailing understanding of systems biologists is to think of pathways and processes as warm and fuzzy clouds: warm since their representation is close to the truth but not necessarily exact; fuzzy since the membership of components in a pathway is graded and dynamic, and therefore not all components of a pathway are equally important and might vary; and a cloud since the boundaries of a pathway are not sharply defined because, among other reasons, many of the pathways and processes connect to form a network [10]. This new definition has inspired the development of novel algorithms that incorporate network statistics to derive biological knowledge from molecular profiles. Two important steps can be discerned: (1) network inference, which is the process of building networks from molecular data [11], and (2) network enrichment analysis, which incorporates topological information present in the network to identify which pathways and processes are relevant and how they are associated with each other in the context of the network. Network analysis will enable researchers to develop a more holistic view of cell signaling patterns and their interactions [12].

At present, it is important to introduce two conceptually different approaches to derive biological meaning from molecular profiles. Therefore, consider a gene expression profile from cancer cells treated with a ligand (e.g., vascular endothelial growth factor). The gene expression profile can be regarded as a functional read-out from a set of pathways that are activated upon ligand-receptor interaction and that culminate in the activation of transcription factors, which cause expression changes. Methods to reveal these upstream signaling pathways based on expression changes exist and are henceforth termed “bottom-up” approaches. In turn, expression changes will endorse a biological response, for example the induction of angiogenesis. Methods to translate gene expression changes, or any other molecular profile, into downstream biological responses are henceforth termed “top-down” approaches.

The top-down approach is essentially initiated on the identified molecular profile (e.g., the expression profile identified in the imaginary experiment described above). The list of genes then goes through a sequence of (1) network inference, (2) network topology analysis, (3) pathway identification through GSEA, and (4) pathway prioritization using network and pathway statistics identified in steps 2 and 3. Pathway mutual exclusivity and co-occurrence, revealed by evaluating overlaps between sets of pathway-specific genes, may provide extra guidance during pathway prioritization [13]. In the bottom-up approach, the same sequence is used, but the genes on which the sequence is initiated represent the set of transcription factors identified through target gene enrichment analysis, thus the transcription factors that drive the molecular profile. Prior to target gene enrichment analysis, gene clustering can assist in finding sets of co-regulated genes, and identifying transcription factors based on co-regulated target gene expression may lead to more biologically meaningful results [14].

With respect to the analysis strategies outlined above, four remarks should be made. First, intuitively, the bottom-up approach is better for identifying pathways, whereas the top-down approach is more appropriate to evaluate biological processes. Nevertheless, the top-down approach performs equally well in identifying signal transduction pathway activation secondary to the prior molecular changes. Therefore, these two approaches should not be regarded as mutually exclusive with respect to the pathway-process distinction. Second, networks are extremely suited for data integration [12]. Therefore, the outlined analysis strategies can be used to perform pathway analysis based on multiple molecular profiles (e.g., mutational and expression data). The dynamic properties of signaling networks (e.g., feed-back and feed-forward loops) can be visualized, for example by incorporating gene expression fold-changes during the network analysis stage. Similarly, contributions of different cell types to the signaling network can be evaluated by supplying metagene expressions that can be regarded as expression contributions of independent cell populations that co-exist in a tumor and that can be identified through multidimensional scaling techniques, such as principal component analysis. Relevant network interactions can then be analyzed using hidden Markov models or conditional random field models [15]. Third, although the bottom-up approach can be applied to all molecular profiles, the description provided in the previous paragraph is only truly applicable to transcriptional profiles, due to the integration of the target gene enrichment analysis step. Last, deciphering tumor biology from molecular profiles critically depends on the quality of the tissue sampling procedure. From this perspective, the “garbage in, garbage out” computer science principle, which refers to the fact that computers will process unintended input data to produce nonsensical output, is also applicable in this setting. Several technical aspects that may have deleterious effects on data quality should be considered, including ischemia and fixation time, used fixatives, and potential sampling bias. No matter how sophisticated the analysis pipeline is, such quality issues cannot be resolved. Thus, researchers who are developing cancer profiling studies should focus primarily on establishing a rigid tissue sampling protocol. In addition, the tissue sampling protocol could also take into account tumor heterogeneity, thus providing multiple samples from the same tumor for molecular analysis. This may enable researchers to analyze in detail the different biological processes and signal transduction mechanisms operational in one tumor sample; this may also enable them to understand how the integration of these signals drives tumor biology, such as metastatic progression and therapy resistance.

Finally, the outlined analysis strategy does not represent a novel algorithm. Rather, it presents an analysis philosophy that builds on already existing tools, most of which are freely accessible online, such as BioConductor and R packages, Cytoscape plugins, and Java tools like Expression2Kinases [14].
